# Label-free multimodal imaging of infected *Galleria mellonella* larvae

**DOI:** 10.1038/s41598-022-24846-7

**Published:** 2022-11-27

**Authors:** Elsie Quansah, Anuradha Ramoji, Lara Thieme, Kamran Mirza, Bianca Goering, Oliwia Makarewicz, Astrid Heutelbeck, Tobias Meyer-Zedler, Mathias W. Pletz, Michael Schmitt, Jürgen Popp

**Affiliations:** 1 Institute of Physical Chemistry (IPC) and Abbe Center of Photonics (ACP), Friedrich-Schiller-University Jena, Helmholtzweg 4, 07743 Jena, Germany; 2 Leibniz Institute of Photonic Technology, Member of Leibniz Health Technologies, Member of the Leibniz Centre for Photonics in Infection Research (LPI), Albert-Einstein-Straße 9, 07745 Jena, Germany; 3 Jena University Hospital, Center for Sepsis Control and Care (CSCC), Friedrich-Schiller-University Jena, Am Klinikum 1, 07747 Jena, Germany; 4 Jena University Hospital, Institute of Infectious Diseases and Infection Control, Friedrich-Schiller-University Jena, Am Klinikum 1, 07747 Jena, Germany; 5 Jena University Hospital, Leibniz Center for Photonics in Infection Research, Friedrich Schiller University Jena, 07747 Jena, Germany; 6 ena University Hospital, Institute for Occupational, Social, and Environmental Medicine, J, Friedrich-Schiller-University Jena, Am Klinikum 1, 07747 Jena, Germany

**Keywords:** Optical imaging, Multiphoton microscopy, Biofilms, Bacterial infection, Biophotonics

## Abstract

Non-linear imaging modalities have enabled us to obtain unique morpho-chemical insights into the tissue architecture of various biological model organisms in a label-free manner. However, these imaging techniques have so far not been applied to analyze the *Galleria mellonella* infection model. This study utilizes for the first time the strength of multimodal imaging techniques to explore infection-related changes in the *Galleria mellonella* larvae due to massive *E. faecalis* bacterial infection. Multimodal imaging techniques such as fluorescent lifetime imaging (FLIM), coherent anti-Stokes Raman scattering (CARS), two-photon excited fluorescence (TPEF), and second harmonic generation (SHG) were implemented in conjunction with histological HE images to analyze infection-associated tissue damage. The changes in the larvae in response to the infection, such as melanization, vacuolization, nodule formation, and hemocyte infiltration as a defense mechanism of insects against microbial pathogens, were visualized after *Enterococcus faecalis* was administered. Furthermore, multimodal imaging served for the analysis of implant-associated biofilm infections by visualizing biofilm adherence on medical stainless steel and ePTFE implants within the larvae. Our results suggest that infection-related changes as well as the integrity of the tissue of *G. mellonella* larvae can be studied with high morphological and chemical contrast in a label-free manner.

## Introduction

Research in pathophysiology reveals that animal models have contributed substantially to the field of scientific study^1^. Model organisms provide valuable insight into disease processes, and vertebrates in particular have been the gold standard for in vivo studies due to their ability to predict human physiology as well as infection^2^. Vertebrates have been extensively used in the study of various infectious diseases such as pneumonia^3^, urinary tract infections^4^, and sepsis^5^. These models are often used due to their high similarity to humans in terms of body temperature, metabolism, and immune response. However, most of these models have some drawbacks, such as cost, practicality, time constraints, and most importantly, requiring ethical consideration^6^. Due to these limitations, alternative invertebrate model organisms have been introduced that are cheaper, more convenient, readily available, and have fewer ethical restrictions. The greater wax moth (*Galleria mellonella*) and the fruit fly (*Drosophila melanogaster*) have emerged as promising alternative model organisms in several research areas, including pathogenesis, bacterial and fungal virulence, immune response, viral infections, toxin research, and antimicrobial drug testing^7–13^. In contrast to other insect models, *Galleria mellonella (G. mellonella)* is able to grow at a wide range of temperatures, from 37 up to 41 °C, hence allowing the analysis of human pathogens under physiologically relevant conditions^14,15^. This is important because temperature is a crucial factor for the virulence of many pathogens and, hence, microbial pathogenesis^15^.

One peculiar feature of insects, in general, is the resemblance of their innate immune response mechanisms (comprised of cellular and humoral defenses) to human innate immunity. Cellular innate immune responses such as phagocytosis of pathogens by hemocytes have been reported to function similarly to human phagocytes (neutrophils and macrophages)^16–19^.

This feature of immunity makes *G. mellonella* a suitable model for research involving infection-related responses. Several studies have already indicated *G. mellonella* as a successful infection model. For instance, Pereira et al. have elucidated the recent advances of this model to study immune responses against human pathogens^20^. The hematoxylin and eosin (HE) staining process has been previously used to investigate gut damage in *G. mellonella* larvae which were forced-fed by indomethacin, a non-steroidal anti-inflammatory drug, causing gastric ulceration and damage^21^.

Traditionally, the methodologies used for the study of insect models are electron microscopy, brightfield microscopy, and confocal fluorescence microscopy, along with histological staining for biopsies. These are mainly to observe the morphological structure of organs, infection-related infiltration of cells, and bacterial colonization^6,7,21^. Additionally, insect-implant models have mainly been investigated via computer tomography and/or electron microscopy^22^.

One of the challenges faced during the use of conventional fluorescence microscopy or electron microscopy is the tedious procedure of sample handling. Other methods for obtaining biochemical information, such as western blotting, flow cytometry, or gene sequencing, are either time and cost-inefficient, or they necessitate prior knowledge of the biochemicals of interest^23^.

Non-linear microscopy techniques such as coherent anti-Stokes Raman scattering (CARS), two-photon excited fluorescence (TPEF), and second harmonic generation (SHG) have exceeded the aforementioned technologies as they offer unique insight into morphological structures while simultaneously providing chemical contrasts. Fluorescence lifetime imaging (FLIM) also offered additional information to the standard intensity fluorescent images by highlighting different tissue sections based on their different lifetimes. FLIM has particularly been useful in distinguishing between fluorophores with overlapping spectral characteristics^24^ as well as being sensitive to molecular interactions and microenvironments based on the intrinsic fluorescent properties of the sample^25^. These advantages have made FLIM desirable for investigating cell metabolic changes^26,27^, highlighting infection-related changes^28^, and analyzing bacterial membranes interacting with *G. mellonella* apolipophorin III^29^.

These optical techniques have gained the upper hand in disease diagnostics and have proven to be faster, along with generating high-throughput morpho-chemical data without the need for external labels^30–36^. They have also been extensively used in human and animal models to study lipids, NAD(P)H, and collagen, all of which are well-known molecular markers for describing inflammation^30–32,35^. In recent studies, there has been an increase in the number of research contributions focusing on insects’ immune responses using the aforementioned molecular markers^36–39^.

Herein, label-free multimodal methods are presented as an effective tool for morpho-chemical investigations of tissue architecture and pathogenesis in planktonic-and biofilm-associated bacteria-infected *G. mellonella* larvae. In this case, two different kinds of bacterial species were used for systemic and biofilm infection due to the different virulence profiles of gram-positive and gram-negative bacteria in the *G. mellonella* larvae. The first part of the study focuses on planktonic infection by the administration of increasing concentrations of gram-positive bacteria such as *Enterococcus faecalis* (*E. faecalis*) into the larvae. The second part of the study addresses the use of a biofilm-associated infection model using gram-negative bacteria such as *Pseudomonas aeruginosa* (*P. aeruginosa*), which are highly virulent in *G. mellonella* larvae. The biofilm-associated infection model was generated by inserting two different strains of *P. aeruginosa*-infected stainless steel and expanded polytetrafluoroethylene (ePTFE) implants into the larvae. The changes due to pathophysiological consequences arising from the strong and vigorous bacterial infection were exploited using multimodal methods, namely, fluorescence lifetime imaging (FLIM), together with CARS, TPEF, and SHG, which were implemented as non-invasive and label-free techniques. FLIM was correlated with conventional histological HE staining for comparison of both visualization methods. Infection-induced damage led to a change in the lifetime of some biomolecules, likely due to the loss of internal structures in the larvae. The studies show the possibility of visualization of the implants inserted into the larvae and ex vivo visualization of the biofilm and biofilm-associated infection in intact tissue.

Overall, we aimed to show the applicability of label-free multimodal imaging methods for a broad range of infection types (i.e., planktonic and biofilm infections) in *G. mellonella* larvae. To the best of our knowledge, this is the first time label-free multimodal imaging techniques have been used in the investigation of *G. mellonella* infection and the visualization of implants.

## Results

### Morpho-chemical information from tissue sections

**Figure 1:**
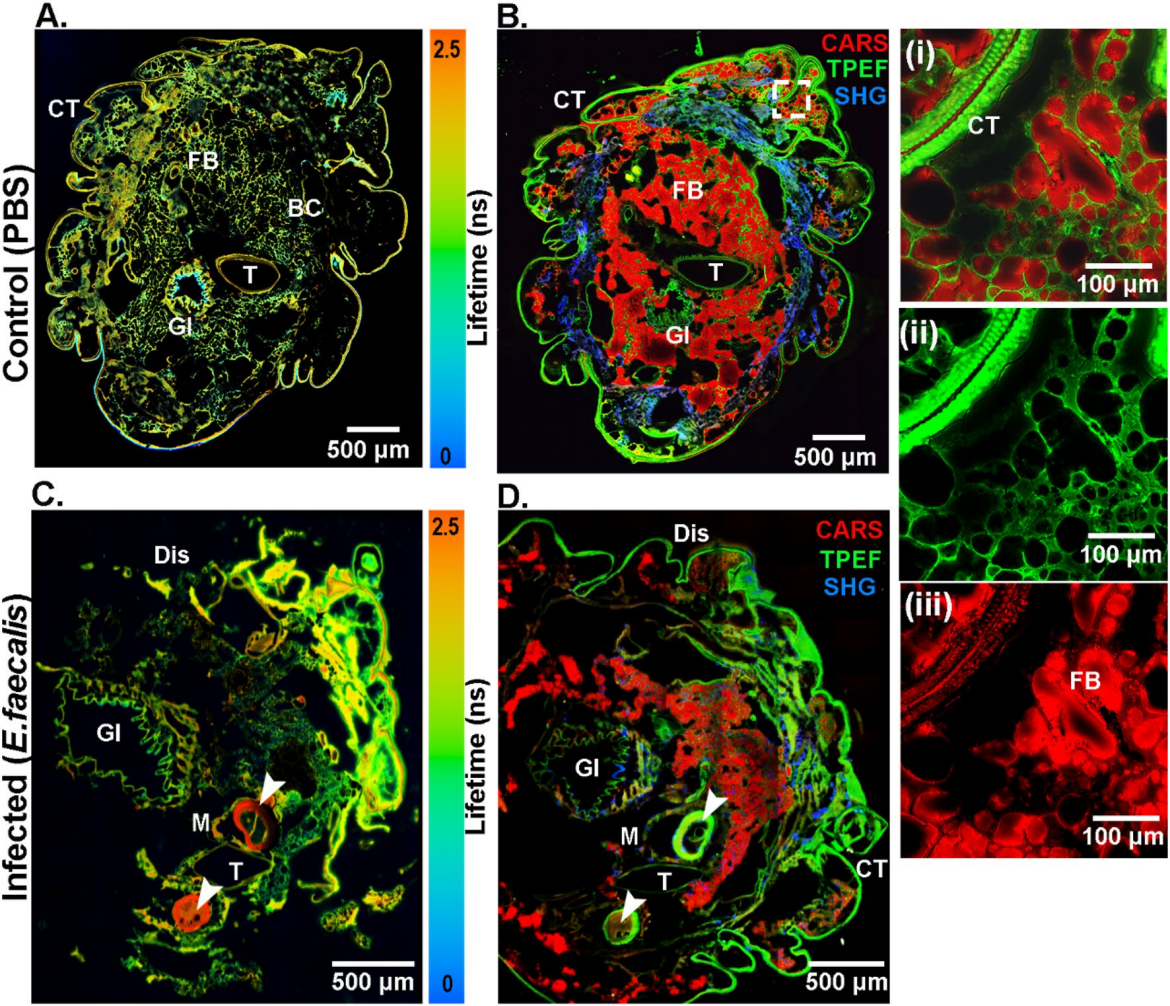
Multimodal images of transverse sections of control (**A**,**B**) and *E. faecalis* infected (**C**,**D**) tissues from *G. mellonella* larvae highlight the tissues’ morpho-chemical information. (**A**,**C**) The FLIM images illustrate transverse sections corresponding to endogenous fluorophores such as flavins and lipo-pigments with differing lifetime information as false-color images ranging from blue to red (scale 0–2.5 ns). (**B**,**D**) The CARS (red), TPEF (green), and SHG (blue) channels were merged as indicated in false colors. The square inset with dashed lines in (**B**) was zoomed and shown in the composite image (**i**), the TPEF channel in green (**ii**), and the CARS image in red (**iii**). M = dislocation of the membrane around tubular organs, Dis = distorted cuticle structure, BC = body cavity, FB = fat body, CT = cuticle, T = trachea, GI = denotes the lumen of the gastrointestinal tract, and white arrowheads point to silk glands.

The transverse sections of the *G. mellonella* larvae, both non-treated and infected by *E. faecalis,* were analyzed in a label-free manner using multimodal imaging combining FLIM, CARS, TPEF, and SHG. By applying FLIM, different body and tissue compartments, including tubular organs such as silk glands, gastrointestinal tract (GI), and body cavity (BC), could be visualized (Fig. 1A,C). For FLIM imaging, a lambda scan was also performed over a range of wavelengths from 489 to 780 nm to detect the endogenous fluorophores in the tissue sections (Supplementary Figure S1). Fluorescent emission spectra were acquired from different regions of interest (ROIs) in the tissue, such as the crypt of the gastrointestinal tract (550 nm), the silk gland (570 nm), the tracheal wall, autofluorescence from the fat body (570 nm), and the cuticle (540 nm). The emission peaks towards 540 nm and 570 nm can be attributed mainly to flavins and lipo-pigments^40–42^. FLIM imaging, being label-free, provides biochemical information about the molecules in their native state. For example, the biomolecules in the non-treated *G. mellonella* larvae (Fig. 1A) showed a lower lifetime in certain regions compared to the infected *G. mellonella* larvae (Fig. 1C). Moreover, the biomolecules comprising the fat body (FB) and the cuticle of both non-treated and treated *G. mellonella* larvae have longer lifetimes, which can be attributed to lipo-pigments and flavins. This can especially be seen in Fig. 1C, where the silk glands show the presence of biomolecules with the highest lifetime, corresponding to lipo-pigments (red color, marked with white arrowheads).

CARS was applied to visualize the lipid distribution in the fat body (FB) and fat tissues as pseudo-colored in red (Fig. 1B,D). The TPEF channel prominently visualized the autofluorescence signal on the outer surface and most internal organs, such as the gastrointestinal tract (GI) and the trachea (T). In Fig. 1B,D, the autofluorescence signal surrounding the FB, the cuticle (CT), and the tracheal wall (T) were observed in green. In the inset, a zoomed composite image (Fig. 1Bi**)** of the fat body (CARS signal, Fig. 1Biii) and autofluorescence of the connective tissue (TPEF signal, Fig. 1Bii) have been highlighted.

In the TPEF images shown in Fig. 1B,D, other morphological structures such as the trachea, lumen, and crypts can be visualized, as well as the silk glands (marked with white arrowheads), which are more prominently visible in Fig. 1C,D. In the non-treated *G. mellonella* larvae, the basement membrane (in green) and the cuticles were intact (Fig. 1B). However, morphological changes were observed in the infected sample (Fig. 1D). The normal, intact body shape was massively deformed. The epidermal cuticle integrity was compromised, resulting in a wrinkled and distorted structure. There was also a detachment of the cuticle from certain regions of the larval body (labeled as Dis) and a partial dislocation of the membrane around the tubular organs (M).

### Pathophysiological consequences due to infection in the *G. mellonella* larvae

A varied response was triggered in the *G. mellonella* larvae upon administering *E. faecalis* bacteria (a concentration of up to 10^6^ CFU/larvae), leading to pathophysiological consequences that were imaged with the label-free multimodal imaging methods. To access these changes, FLIM was used to generate false-color fluorescence lifetime images in comparison to HE-stained images (see Fig. 3).

**Figure 2:**
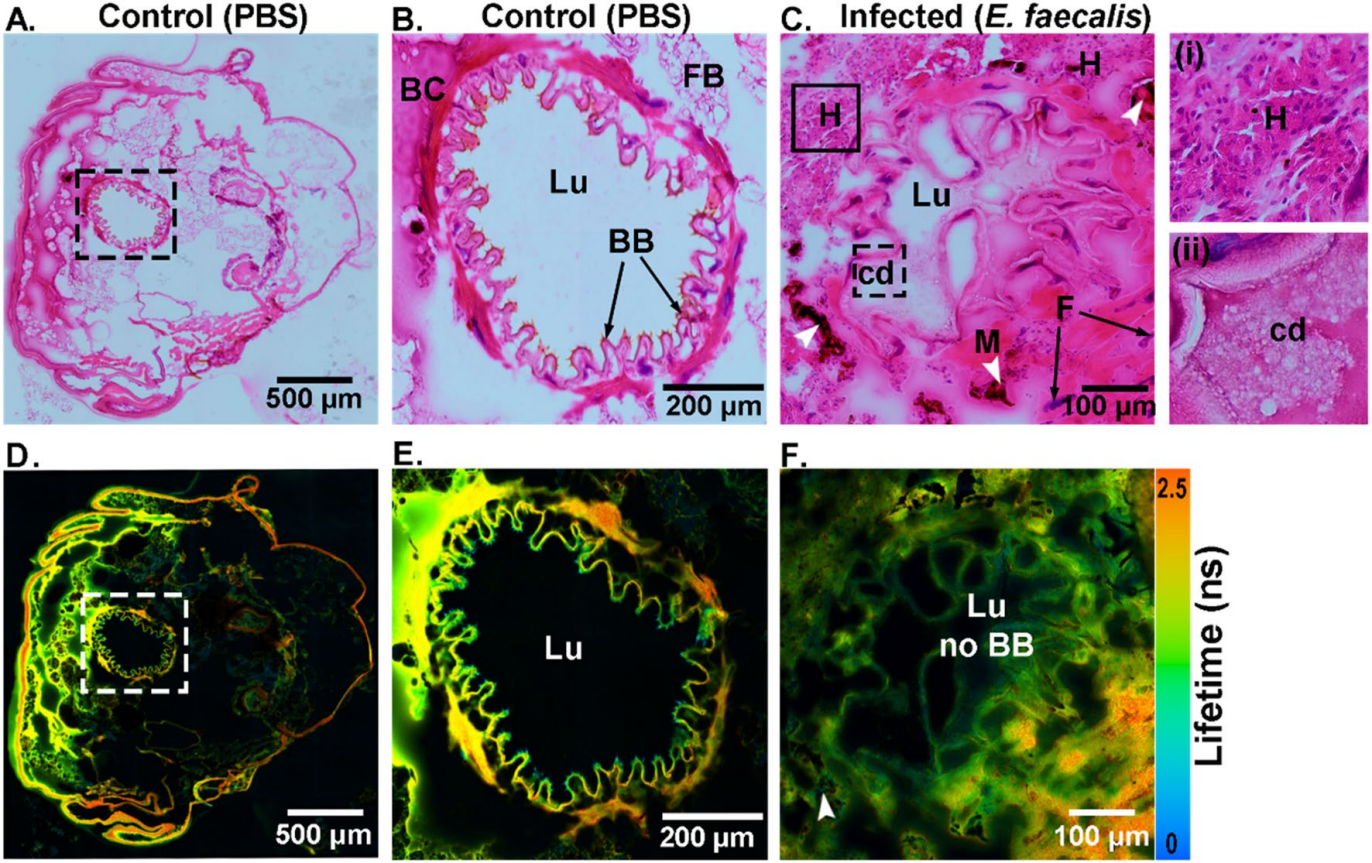
Visualization of the transverse section of the control (**A**,**D**) and *E. faecalis* infected (**C**,**F**) tissue from *G. mellonella* larvae. (**A–C**) HE stained images of tissue sections. (**D–F**) FLIM images illustrate transverse sections corresponding to endogenous fluorophores such as flavins and lipo-pigments with differing lifetime information as false-color images ranging from blue to red (scale 0–2.5 ns). The squared-dashed sections in (**A**,**D**) have been zoomed-in and are shown in (**B**,**E**), respectively. The squared inset with a solid line in (**C**) is zoomed-in and shown in (**i**), and the squared inset with a dashed line in (**C**) is shown in (**ii**). BB = brush border, Lu = lumen, FB = fat body, BC = body cavity, cd = cell damage, F = cytoplasmic fragmentation, M (white arrowheads) = melanization, H = areas with hemocyte infiltration.

**Figure 3:**
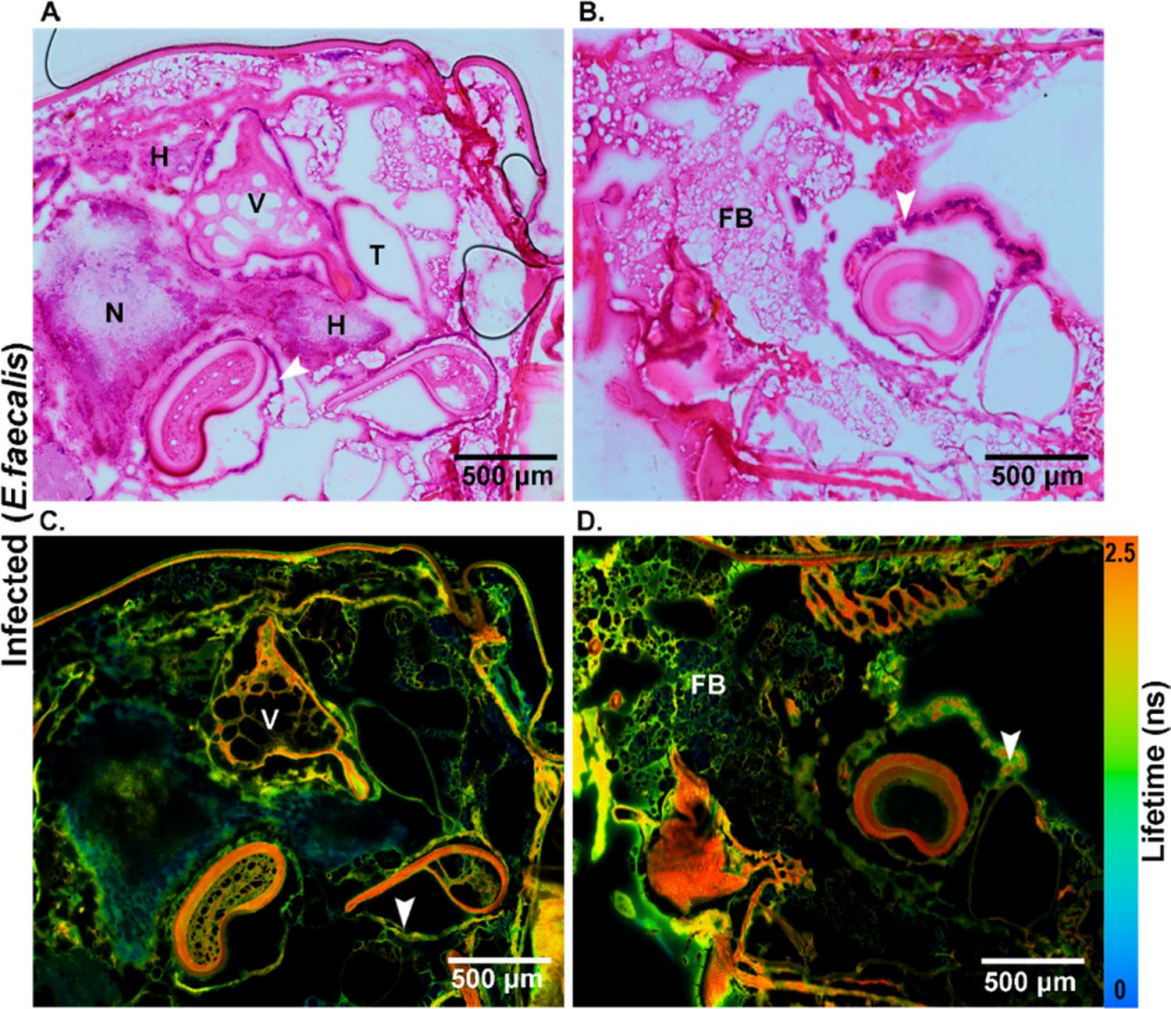
Visualization of the transverse section of *E. faecalis* infected tissue from *G. mellonella* larvae. (**A**,**B**) HE stained images and (**C**,**D**) FLIM images illustrate endogenous fluorophores such as flavins and lipo-pigments with different lifetime information as false-color images ranging from blue to red (scale 0–2.5 ns). V = vacuolization, N = nodules, FB = fat body, H = hemocytes, T = trachea. White arrowheads indicate the dissociation of the tissue around tubular organs or silk glands.

The control sample in the presence of PBS did not show any indications of tissue deterioration (Fig. 2A,D). In the infected samples, however, the loss of the brush border (BB) within the larvae’s gut was visible (Fig. 2C,F), whereas in the control sample the BB was intact, as can be seen in the zoomed images shown in Fig. 2B,E. As a defense mechanism of *G. mellonella* larvae against bacteria, melanization (M, marked as white arrowheads), or blackening of the tissues caused by phenoloxidase (PO) activity, could be visualized in Fig. 2C,F. The localization of several bacterial colonies in the tissue that surrounded the tubular organs was visualized and confirmed through the HE images. Supplementary Figure S2 shows a similar occurrence, as indicated by the white arrowheads. In addition, cytoplasmic fragmentation (F), hemocyte infiltration (H) (see Fig. 2(i)), cellular damage (cd) (see Fig. 2(ii)), and partial displacement of gut lining into the lumen were indications of altered tissue morphology. Further changes due to infection-induced damage were observed in Fig. 3A,C.

The cavities/voids in a tissue, known as vacuolization, were visualized in the FLIM image and confirmed in the HE-stained image. This phenomenon usually occurs after a bacterial infection in the host, causing the death of many cells^43^. Apart from vacuolization (V), there was also dislocation of the membrane around the tubular organs (marked as white arrowheads). Bacterial colonies were found to be condensed in those regions (Fig. 3B,D). Along with the infiltration of hemocytes was the formation of nodules, which was evidenced as a cellular defense mechanism (Fig. 3A). These inflammatory nodules indicated the presence of basophilic bacterial colonies, as indicated by the white arrowheads (Fig. 3B,D).

### Multimodal visualization of the biofilm formation on implants in vitro and ex vivo

**Figure 4:**
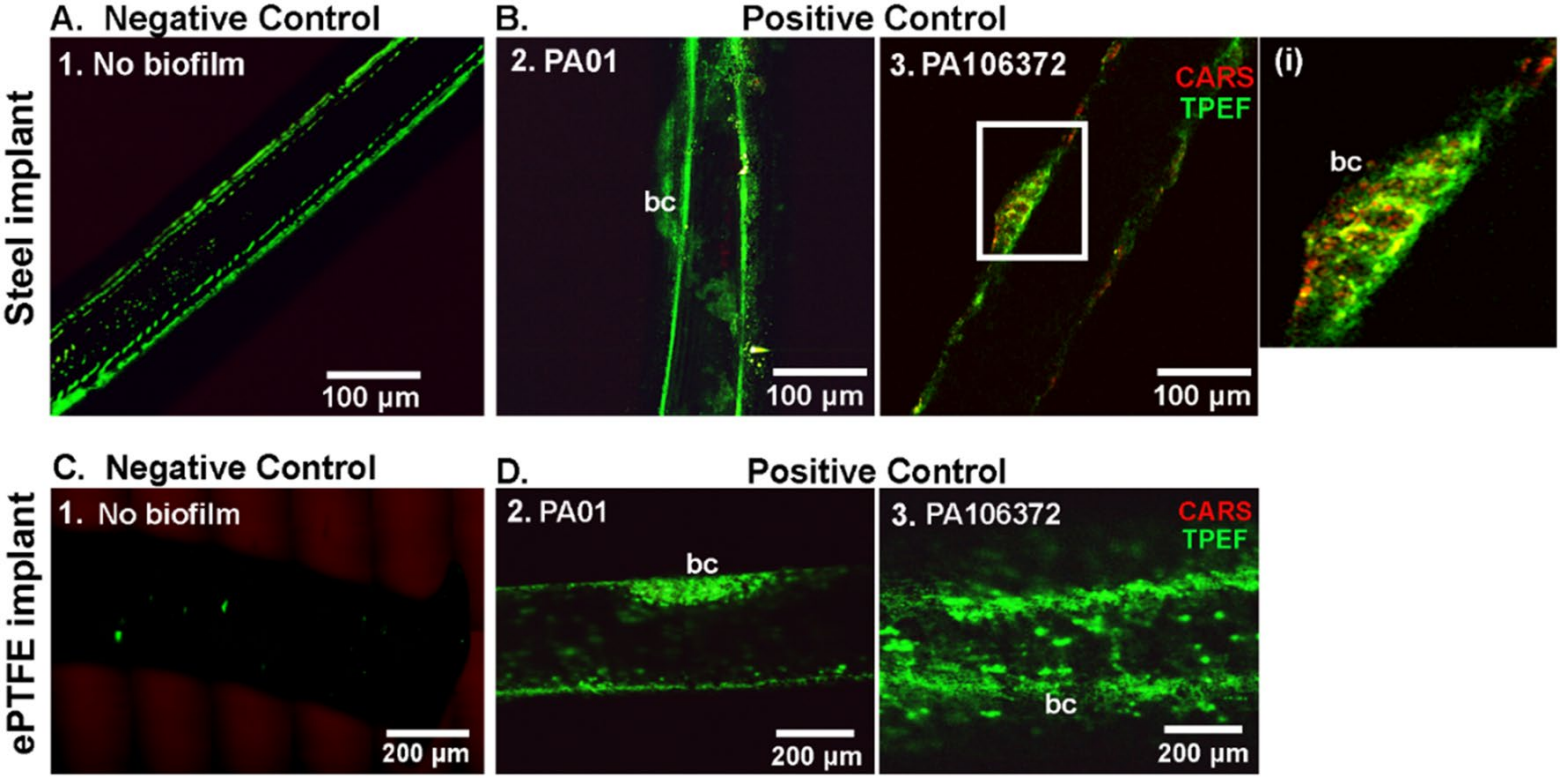
Multimodal images of the implants with biofilm formed in vitro by *P. aeruginosa* bacterial strains PA01 and PA106372. The steel implants were without biofilm (**A1**) and with biofilm formed by the bacterial strains PA01 (**B2**) and PA106372 (**B3**). The square inset with a solid line in (**B3**) is zoomed in and shown in (**i**). Bacterial clusters (bc) formed by the bacterial strains PA01 (**D2**) and PA106372 (**D3**) were observed on the ePTFE implant. Multimodal images with CARS (red) and TPEF (green) channels are overlayed in false colors.

In the previous part of this work, an inflammatory response triggered by the planktonic bacterial infection induced by *E. faecalis* in *G. mellonella* was investigated. The following sections will highlight biofilm-associated infection after administering *P. aeruginosa*-infected implants to *G. mellonella* larvae. *P. aeruginosa* is a classical biofilm-associated pathogen, which makes it suitable for evaluating the detrimental consequences associated with implants with biofilms. This part of the study employed CARS/TPEF/SHG to visualize the biofilm formed in vitro by two strains of *P. aeruginosa* bacteria (PA01 and PA106372) on implants (steel and ePTFE), as shown in Fig. 4.

The implants without biofilm (Fig. 4A1,C1) are referred to as negative (non-infected) controls, and the implants with biofilm (Fig. 4B,D) are called positive (infected) controls. The multimodal imaging was comprised of only CARS and TPEF imaging as no SHG signal was detected. In addition, the FLIM method was also applied to visualize the biofilm on the implants, as shown in Supplementary Figures S3 (steel implant) and S4 (ePTFE implant). Two images per implant with biofilm imaged at different parts of the implant are shown. In the CARS/TPEF-multimodal images, the negative control steel implant showed no indication of biofilm formation (Fig. 4A1). In the CARS channel, the implants appeared as dark outlines and as green rectilinear structures in the TPEF channel (Fig. 4A1).

No CARS signal was observed in the plastic implant, although contributions from the ePTFE material itself can be detected in the CH stretching region. The lack of CARS signal is because the bands around 2870 cm^−1^ are very weak in intensity^44^ and are comparable to the vibrational band (2850 cm^−1^) probed within this study. For the infected implants, noticeable clusters of bacteria were identified around the positive control steel implant (Fig. 4B), which was mostly resolved by the TPEF signal with punctuated CARS signal in red (as shown in the inset image Fig. 4i). The biofilm matrix is usually composed of extracellular polymeric substances, mostly polysaccharides, protein components, nucleic acids, and lipids^45^. In this instance, the CARS signal tuned at 2850 cm^−1^ is sensitive to the lipid component of the biofilm.

For the ePTFE implants (Fig. 4C,D), similar results were observed, except for the weak fluorescence signal from the ePTFE implant, which was observed in the negative control (Fig. 4C1). Moreover, the CARS signal observed in the biofilm matrix on the steel implant (Fig. 4B3) is missing in the plastic implant (Fig. 4D3). The technical cause of this occurrence is the forward-generated CARS signal, which is back-reflected from the stainless-steel surface and focused on the detection optics, therefore increasing the total number of scattered photons to be detected.

To ascertain the CARS/TPEF visualization of the bacterial biofilm formation on the implants, pure bacterial suspensions of *P. aeruginosa* strain (PA01), both in water (Supplementary Figure S5A1) and in dried form (Supplementary Figure S5A2), were imaged at a higher magnification. In the CARS and TPEF channels, single bacteria were resolved and visualized as shown in the supplementary figure (Supplementary Figure S5). The autofluorescence of the bacterial pigment pyocyanin was mainly detected in the TPEF channel.

Further, FLIM imaging complemented the observations made by CARS/TPEF via changes in their lifetime information with the presence or absence of the biofilm (Supplementary Figures S3 and S4). Based on the FLIM images, a lower lifetime (depicted by blue areas) of the negative control steel implant without biofilm (Supplementary Figure S3A) was observed in comparison to the long lifetime information from the positive controls (Supplementary Figure S3B). The change in lifetimes suggests bacterial clusters and biofilm attachment to the steel. Due to the weak fluorescence, no FLIM image was obtained for the negative ePTFE control implants (Supplementary Figure S4A). However, differing lifetimes, ranging from blue to red, were observed for positive ePTFE control implants (Supplementary Figure S4B). FLIM images of the pure bacterial suspensions of *P. aeruginosa* strain (PA01), both in water (Supplementary Figures S5B3 and S5B5) and in dried form (Supplementary Figures S5B4 and S5B6), were also taken. The *P. aeruginosa* bacteria could be visualized at a single-cell level, and high lifetime values were indicative of the pyocyanin pigment, marked with white arrowheads (Supplementary Figure S5).

**Figure 5:**
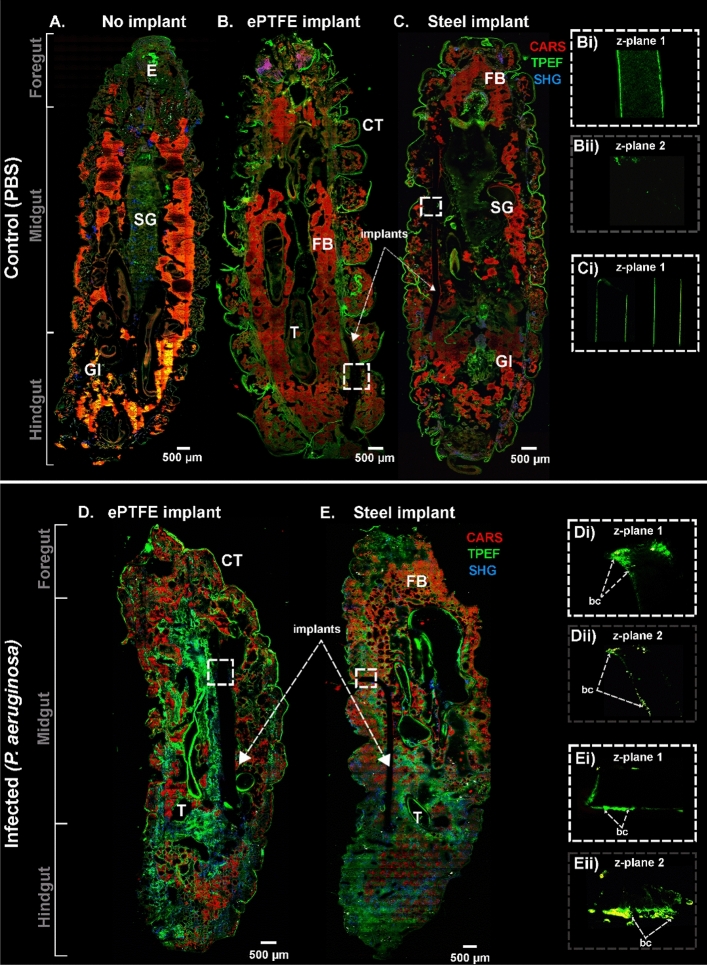
Multimodal images showing the sagittal section of the *G. mellonella* larval body displaying the entire larvae of control without an implant (**A**), with PBS-incubated ePTFE (**B**) and stainless steel (**C**) implants. *G. mellonella* with *P. aeruginosa* (PA01)-infected ePTFE (**D**) and stainless steel (**E**) implants. Multimodal images with CARS (red), TPEF (green), and SHG (blue) channels are overlayed in false colors. The square inset with white dashed lines was zoomed from (**B**,**C**) to visualize parts of the plastic implant in (**Bi**) and the stainless-steel implant in (**Ci**), respectively. The image shown in (**Bii**) is of the same implant as shown in (**Bi**) but acquired at a different focus depth (gray dashed lines) along the z-axis (z-plane 1 and z-plane 2) of the implants. Similarly, the square inset with white dashed lines was zoomed from (**D**,**E**) to visualize parts of the plastic implant in (**Di**) and the stainless-steel implant in (**Ei**), respectively. The images shown in (**Dii**,**Eii**) are of the same implants as shown in (**Di**,**Ei**), respectively**,** but acquired at a different focus depth (gray dashed lines) along the z-axis (z-plane 1 and z-plane 2) of the implants. FB = fat body, CT = cuticle, T = trachea, GI = gastrointestinal tract, SG = silk gland, E = esophagus, bc = bacterial cluster.

**Figure 6:**
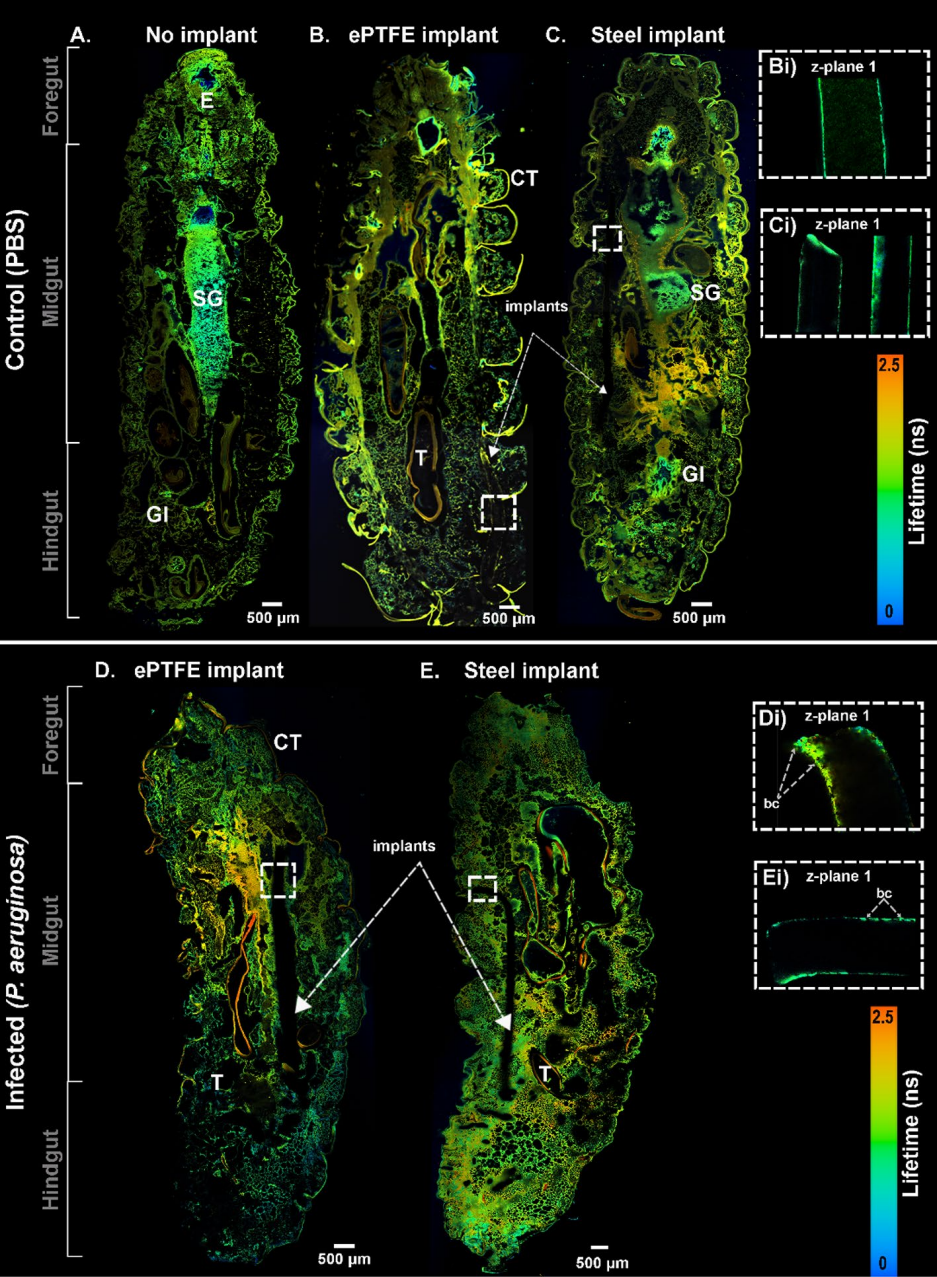
A FLIM image showing the sagittal section of the *G. mellonella* larval body displaying the entire larvae of control without an implant (**A**), with PBS-incubated ePTFE (**B**) and stainless steel (**C**) implants. *G. mellonella* larvae with *P. aeruginosa* (PA01)-infected ePTFE (**D**) and stainless steel (**E**) implants. The differing lifetime information from endogenous fluorophores such as flavins and lipo-pigments is presented as false-color images ranging from blue to red (scale 0–2.5 ns). The squared insets with white dashed lines in (**B–E**) were zoomed to visualize the plastic implant at a different focal depth (z-plane 1) in (**Bi**,**Di**) and the stainless-steel implant in (**Ci**,**Ei**), respectively. CT = cuticle, T = trachea, GI = gastrointestinal tract, SG = silk gland, E = esophagus, bc = bacterial cluster.

In Fig. 5, the sagittal tissue section of *G. mellonella* larvae with the *P. aeruginosa* (PA01) infected implants is shown in the CARS, TPEF, and SHG images. In addition to this, the co-registered FLIM images, as illustrated in Fig. 6, were acquired on the same larvae with implants to complement the multimodal imaging results. The control larvae without any implants are shown in Figs. 5A and 6A. However, the data for the *P. aeruginosa* bacterial strain PA106372 is not shown. Here, the *P. aeruginosa* (strain PA01) larvae with an infected ePTFE implant (Fig. 5D; also see zoomed images in Fig. 5Di,Dii) and a stainless-steel implant (Fig. 5E; also see zoomed images in 5Ei and 5Eii) can be visualized. The images capture the entire larvae, displaying the foregut, midgut, and hindgut. The CARS channel, shown in red, captures the fat body, which is seen as many lobes distributed throughout the larval body.

Further, to ascertain visualization of the biofilm on the implants, negative control samples of PBS-treated steel and ePTFE implants (Fig. 5B,C; also see zoomed images in Figs. 5Bi,Bii,Ci; Fig. 6B,C; also see zoomed images in Fig. 6Bi,Ci) were analyzed and compared to the infected implants. The control PBS-treated implants in the larvae show no indication of adherence (such as hemocytes, plasmatocytes, or granulocytes) to the implants, indicating that the signals observed on the implants in infected larvae are from the biofilms. The presence of biofilm is confirmed further by comparing the multimodal/FLIM signals of biofilm from in vitro implant samples (Fig. 4 and Supplementary Figures S3 and S4) to biofilm from ex vivo implant samples (Figs. 5D,E and 6D,E), which produce very similar results.

In addition to the single photon FLIM analysis, 2P-FLIM images were taken to confirm the presence of NAD(P)H in the tissue sections by exciting at 672 nm and detection with a 458/64 nm filter. A 2D-correlation graph of ${\mathrm{t}}_{1}$ and ${\mathrm{t}}_{2}$ was generated using a biexponential fit of the data, illustrating two separate clusters. The strongest cluster is made up of contributions from free (∼ 400 ps) and protein-bound (∼ 1 to 5 ns) NAD(P)H^24^, while the second cluster includes contributions from other fluorophores (Supplementary Figure S6).

## Discussion

In this study, multimodal imaging with the combination of CARS, SHG, and TPEF, along with FLIM and conventional HE staining, was employed for the detection of morpho-chemical changes occurring in healthy and diseased larvae of *G. mellonella*. The key result of the applied methods was the visualization of the larvae’s tissue integrity and pathophysiological consequences arising after planktonic bacterial infection with *Enterococcus faecalis*. Further, we visualized biofilm formed by *P. aeruginosa* bacteria on two different implants, namely, steel and ePTFE. This is the first study where label-free imaging has been applied to visualize tissue deterioration due to the massive infection of *G. mellonella* larvae and the detection of biofilms on the implants within the larvae.

Multimodal imaging allowed us to capture infection-related responses within the larvae, i.e., the infiltration of hemocytes at the site of infection. The hemocyte infiltration leads to nodule formation and melanization of hemolymph, which is intended to limit the infection. The multimodal imaging, both spatially and with high chemical contrast, resolved the nodule formation and melanization as shown in Fig. 3. Further tissue alterations, such as vacuolization and cytoplasmic fragmentation, follow the aforementioned pathophysiological consequences. These observations are in agreement with the literature, where mostly labeled and/or sample-destructive methods such as electron microscopy are applied for visualization^16,46–50^. The advantage of the multimodal imaging method is that it is label-free and utilizes chemical contrast, such that infection-relevant changes can be followed during the initiation phase of the disease. Furthermore, multimodal imaging provides the possibility to evaluate off-target changes due to the infection occurring within the larvae. For example, although the intestinal barrier is well conserved within the larvae with the infection within the hemocoel, the multimodal images revealed loss of the brush border of the microvilli, which is the primary site of nutrient absorption. There were indications of gut tissue deterioration, such as a partial displacement of cells and gut lining into the lumen and the formation of bacterial colonies around tubular organs, displacing their membranes. These alterations typically occur when the infection reaches the gut, resulting in a dysbalanced host response towards infection, and have been well documented both in insects and in mammals^51,52^.

Specifically, further insight into the provoked defense mechanism in the larvae can be gained through the endogenous molecular markers that come into play during infection. The infected tissues were further examined to visualize the targeted endogenous molecular markers such as lipids, collagen, and NAD(P)H^35,53^ in a spatially resolved manner. The lipid distribution, as monitored by CARS, was visualized in the adipocytes in the insect’s fat body. The control sample without an implant showed a strong CARS signal (Fig. 5). In comparison to the control larvae inserted with PBS-incubated implants (Fig. 5B,C), the increment in CARS signal was also noticeable. The similarities in these controls indicate no adverse effects of the implants, such as melanization or toxicity, which confirms the biocompatibility of steel and ePTFE implants^22^. Any lipid-related changes that occur during infection are usually a result of the metabolic adaptation of the host. Lipid metabolism can be significantly altered, which, in this case, was observed as reduced lipid content or fatty acids. A detailed view can be seen in the whole larvae images displayed in Fig. 5D,E. Interestingly, amongst the lipid structures were autofluorescent signals, which were resolved by the TPEF channel. The autofluorescent signal around the fat body has also been identified in *Drosophila melanogaster* based on other studies^54,55^. In our case, the TPEF signal was seen to increase, accompanied by a decrease in lipid content (see Fig. 5D,E). The increment in the TPEF signal could also be due to the infiltration of hemocytes as a defense mechanism against infection. The autofluorescence from the TPEF channel observed in the larval tissues can be attributed to NAD(P)H. As reported by Kolenc et al., NAD(P)H fluorescence can be detected between 440 and 470 nm when excited at 330–360 nm for one photon and < 760 nm for two photons^56^. This corresponds to the excitation and detection wavelengths used in this study for TPEF imaging, which is 672 nm and 458 nm, respectively, and are therefore optimum for detecting NAD(P)H. In our previous studies, we were able to detect NAD(P)H autofluorescence within cells and tissues by using the same imaging configuration^35,53^.

According to previous studies, *G. mellonella* larvae have been found to contain proteins that are identical to the human NAD(P)H oxidase complex in neutrophils^57–59^. As stated earlier, we associate the autofluorescence signal in the TPEF channel with NAD(P)H, which is also involved in innate immunity. Autofluorescence signal from NAD(P)H, was detected in various parts of the larval tissue, such as the insect’s cuticle, microvilli, brush border, and some tubular organs. In the case of SHG imaging, the extracellular matrix component of collagen fibers was visible in the muscle tissue and basement membrane regions of the control tissue (Fig. 1B) but was reduced in comparison to the infected tissue observed in Fig. 1D. Even though very few collagen fibers were recorded, this finding is consistent with previous research. As indicated by Adachi et al., collagen IV in insects is involved in innate immunity and demonstrates loss of fibers in inflamed regions^37,60^. Due to the tissue's sagittal cross-sectioning, which obscures the muscular tissue in some regions (Fig. 5), less SHG signal was seen in the control samples than in the infected samples. Moreover, FLIM presented molecular contrast based on the fluorescence lifetime information of endogenous fluorophores such as flavins and lipo-pigments. For instance, the lifetime of the gastrointestinal tract (GI) and tubular organs was longer than that of the cuticle but showed similar intensity levels in traditional fluorescence imaging. FLIM has previously been used in the study of insect organs and tissue metabolism, to achieve deeper penetration into lipid membranes and research into peptides bound to bacteria cells^39,61,62^. FLIM analysis indicated higher lifetime information in the infected tissue regions with bacterial colonies and melanin (tissue blackening), and/or hemolymph clotting.

Although HE-stained images could also identify the infection-related changes and discriminate between the infected and the control samples, molecular characterization and changes due to metabolic activity were not possible. In particular, the rise in lipid distribution in CARS and the reduction of the autofluorescence in the TPEF signal were not recorded in the stained images.

In addition to the structural and physiological studies of the larval tissue, an insect-implant model was presented in this study. We qualitatively demonstrated the possibility of visualizing biofilm formed by two *P. aeruginosa* strains on steel and plastic surfaces using non-invasive methods, as explained in “Multimodal visualization of the biofilm formation on implants in vitro and ex vivo” section. According to our results, the biofilm formed on the implants in the in vitro condition was resolved by the TPEF channel when imaged at a different depth of focus than the larval tissue. This is because the implants are thicker than the tissue section. The strong bacterial TPEF signal was from the pyocyanin pigment (excitation/emission at 360/460 nm^63,64^), known to be produced by *P. aeruginosa*, and this was confirmed by imaging bacterial suspension samples^65,66^. The TPEF signal observed in Fig. 5Di,Dii,Ei,Eii was mainly from the *P. aeruginosa* bacteria, which was confirmed by measuring pure bacterial suspension both in wet and dry conditions (see Supplementary Figure S5). Furthermore, the FLIM images confirmed this finding by providing increasing autofluorescence lifetime information of the positive control implants with biofilm as compared to the significantly lower lifetime of the negative controls.

Overall, the multimodal imaging modalities were capable of assessing the pathophysiological consequences due to infection. They could distinguish between uninfected and infected samples in a label-free manner based on tissue and biofilm characterization. Ultimately, these methods can also serve as a prospective quantitative indicator for biofilm-related infections.

## Conclusion

The results highlighted in this paper show great potential for ex vivo studies and the feasibility of non-invasive, label-free methods for future in vivo investigations in insects and other animal models. CARS, TPEF, SHG, and FLIM have been used to highlight bacterial infection-associated changes in the *G. mellonella* larvae after *E. faecalis* infection. Since immunological studies are an essential part of infection-related diseases, this study serves as a precursor for more advanced research to follow acute and chronic infection-associated morpho-chemical changes in a spatially resolved manner both in insects and mammals. Further findings from this study reveal that they are suitable and reliable surrogate host models for evaluating the morphological and physiological changes before and after infection. The histological HE staining supports and reveals the larva’s internal organs and the disturbed tissue morphology as a consequence of the infection. Moreover, multimodal imaging allows direct visualization of the biofilm formed on the implants, thus enabling the possible study of biofilms and antimicrobial resistance in intact tissue. A better understanding of the *G. mellonella* infection model can aid in the development of therapeutic strategies for the prevention of infection-related complications as well as antibiotic research. Biofilm formation and implant-related infections can be investigated by using an abiotic implant that mimics a medical implant. Further studies can be focused on investigating metabolic changes due to infection as well as on antibiotic research to control biofilm formation and even devise new strategies for antimicrobial-coated medical implants.

## Materials and methods

### Systemic infection of *G. mellonella *with *Enterococcus faecalis*

**Figure 7:**
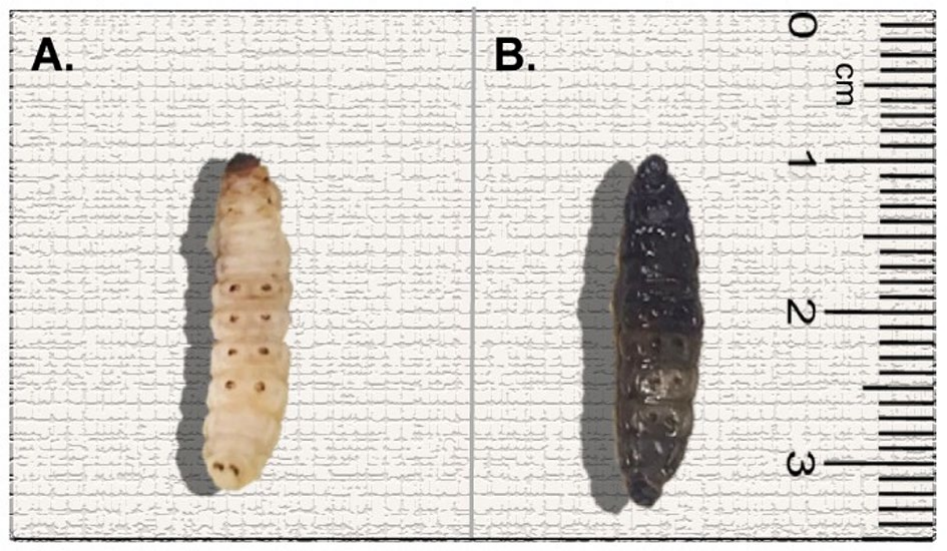
PBS-injected control larva (**A**) and *E-faecalis* infected larva (**B**).

The sample preparation was similar to that reported previously^11^. Briefly, *G. mellonella* wax moth larvae (TruLarv™, BioSystems Technology, Exeter, UK) were infected with a clinical *E. faecalis* isolate (bk1653) via injection of 10  µL of bacterial suspension (10^8^ CFU/mL, corresponding to 10^6^ CFU/10 µL/larva) into the proleg of the larvae with a Hamilton syringe (701N; Merck, Darmstadt, Germany). Control larvae were injected with phosphate buffer solution (PBS). The color change from cream to black was evident after infection with bacteria (Fig. 7). The larvae were incubated at 37 °C and sacrificed 24 h post-infection by freezing at − 80 °C. For microscopy, the frozen larvae were fixed with 4% formaldehyde (Histofix, Roth, Germany) for 1 h and embedded in a Tissue Freezing Medium (Leica Biosystems, Germany). Transverse cryo-sectioning was performed with a tissue thickness of 10 µm. The experiments with the planktonic bacterial infection of the larvae were repeated twice, and in each replication, at least three larvae were used.

### Insertion of steel and ePTFE implants

To model implant-related infections (e.g., cardiac device-related infections, prosthetic joint infections), stainless steel wire (SERANOX^®^) with a thickness of USP 4/0 and polytetrafluoroethylene (ePTFE) suture (GORETEX^®^ SUTURE) with a thickness of CV-4 were used as implant materials. For biofilm formation, the implants were sliced into 0.5 cm sections and each inoculated in triplicates with *P. aeruginosa* strains PA01 and PA106372 in a 96-well microtiter plate with 200 µL of bacterial suspension (0.5 McFarland) per well. The microtiter plate was incubated for 24 h at 37 °C in a shaking incubator. After incubation, the implants were washed twice in 1 × PBS and inserted through the proleg into *G. mellonella* larvae. The larval prolegs were opened by bending the larvae, followed by the vertical insertion of approximately 2–3 mm of the implant. Then, the larvae were relaxed, and the implants were flexed and pushed horizontally into the larvae, in parallel to the gut, under the surface of the cuticula.

Implants incubated in 1xPBS were also implanted into control larvae. Three replicates of infected larvae for each implant and the control larvae were investigated. The larvae were incubated for a further 24 h at 37 °C before being frozen at − 80 °C for microscopy. For the microscopy, the frozen larvae were fixed with 4% formaldehyde (Histofix, Roth, Germany) for 1 h, embedded in distilled water at − 20 °C, and longitudinal cryo-sectioned with a tissue thickness of 10 μm on either a glass slide or coverslip. For the investigation of the implants directly, a similar procedure was followed as mentioned above for the biofilm formation. After the incubation, the implants were washed twice in 1 × PBS and fixed with 4% formaldehyde for 1 h, followed by washing twice with 1 × PBS. The implants were then placed in a petri dish and imaged directly. In addition, a UV-inactivated *P. aeruginosa* (PA01) bacterial pellet was smeared on a glass slide and air-dried for reference measurements.

### Histopathology of larval tissue

The larvae tissue sections after multimodal imaging were stained with hematoxylin and eosin (HE). The tissue sections were dehydrated using an ethanol series of 70%, 80%, and 90% followed by 100% ethanol washes. Dehydrated tissue sections were stained with hematoxylin, followed by washing with distilled water. The hematoxylin-stained tissue sections were further stained with eosin followed by washing in distilled water. The slides were dried in an incubator and stored at room temperature. The brightfield images of HE-stained tissue were collected using an Olympus microscope (Olympus, Germany) with a 40 × /0.8 objective.

### Fluorescence lifetime imaging (FLIM)

For FLIM measurements, the larval tissue sections were analyzed with a Leica TCS SP8 X laser-scanning microscope (Leica Microsystems, Germany) equipped with an internal hybrid photon counting detector, the HyD detector (HyD SMD2), and a pulsed white light laser, which emits from 470 to 670 nm. The pulse repetition rate at 80 MHz ensures that the laser pulses arrive every 12.5 ns and the detector is always in photon counting mode. The fluorescent signal was excited at 488 nm and detected by the hybrid detector in the wavelength range of 498–740 nm using a 20 × microscope objective (HC PL APO CS2, NA 0.75 DRY). Image acquisition was obtained at a pixel resolution of 1024 × 1024 pixels with a pixel dwell time of 1.58 μs. Tile scans of the samples were recorded at a scan speed of 400 Hz with an accumulation of 10 frames and an average of 1 scan per line. LAS X FLIM/FCS software (Leica Microsystems, Germany) was used to perform the analysis on the acquired FLIM data with biexponential decay fittings in a range of 0.0–12.5 ns to fit the fluorescence decay of all pixels in the image pixel by pixel. The varied lifetime components were represented as two-lifetime images corresponding to τ_1_ and τ_2_. The data was adjusted to a threshold of 50 and a binning factor of 1, such that the value of χ^2^ was less than 1.5, which indicates a good fit. The images show false colors with a lifetime range from 0 to 2.5 ns, with blue being the shorter lifetime and red being the longer lifetime.

### Nonlinear multimodal imaging

To investigate additional morpho-chemical information, nonlinear multimodal imaging was employed to highlight chemical constituents of the tissue section, which included lipids for coherent anti-Stokes Raman scattering (CARS), autofluorophores like NAD(P)H in two-photon excited fluorescence (TPEF), and collagen in second harmonic generation (SHG). A detailed description of the experimental setup has been previously described elsewhere^33^. In brief, the combination of CARS, TPEF, and SHG was obtained simultaneously by using near-infrared (NIR) illumination at a wavelength of 832 nm from a Titanium-Sapphire (Ti: Sa) laser (Coherent, Santa Clara, California). The Ti: Sa-laser has an average output power of 3 W at a 76 MHz pulse repetition rate, emitting pulses of 2 ps duration. The output beam is split with a nonpolarizing beam splitter where one part is directly used as the CARS Stokes beam and the other part, termed as the CARS pump beam, is converted by the optical parametric oscillator (OPO, APE, Berlin, Germany) to 672 nm to match the C-H stretching vibration of lipids at 2850 cm^−1^. The two beams are then combined in space and in time by a dichroic filter and a mechanical delay stage, respectively. The combined pump and Stokes beams are subsequently fed into a laser scanning microscope (LSM 510 Meta; Carl Zeiss Microimaging GmbH, Jena, Germany) and focused onto the sample either with a 20 × (NA 0.8) apochromatic objective (Zeiss) for tissue measurements or with a 63 × (NA 1.4, Oil DIC Plan-Apochromat) for implant and bacteria measurements. The CARS signal is detected using a 550 nm bandpass filter, and a 415 nm bandpass filter is used in the detection of the SHG signal. The TPEF signal is collected in the backward direction with the 458/64 nm band pass filter and a 650 nm shortpass filter (Zeiss, Omega, Optics, Semrock, Thorlabs) and detected by photomultiplier tubes (PMT, Hamamatsu Photonics, Hamamatsu, Japan). The measurement parameters include a tile scan with a pixel resolution of 1024 × 1024 pixels, a pixel dwell time of 1.6 μs, and 2 frame averages. Alternatively, to obtain 2P-FLIM images, the fluorescent signal is reflected onto a hybrid GaAsP detector (HPM-100–40, Becker & Hickl, Germany) by a dichroic mirror (600 nm longpass) and filtered by a shortpass 650 nm and bandpass 458∕64 nm (Semrock) filter. For obtaining the fluorescent lifetime, a time-correlated single-photon counting (TCSPC) system (SPC-150, Becker & Hickl, Germany) is used. The imaging parameters for 2P-FLIM measurements include 1024 × 1024 pixels, 1024-time channels, and a pixel dwell time of 1.6 μs.

All the parameters and optical modalities used for the detection of the molecular structure in the larval tissue have been summarized in Table 1.

**Table 1:** Summary of multimodal optical modalities emphasizing molecular contributions detected in tissue^35,40,41,56,67^.

Method	Excitation λ	Detection λ	Molecular contributions
CARS 2850 cm^−1^	Pump: 672 nm Stokes: 832 nm	550 nm	CH_2_/lipids
TPEF 458 nm	Pump: 672 nm	426–490 nm	NAD(P)H
SHG 415 nm	Stokes: 832 nm	415 nm	Collagen
1P-FLIM	488 nm	498–740 nm	Flavins, lipo-pigments
2P-FLIM	Pump: 672 nm	426–490 nm	NAD(P)H

## Supplementary Information

Supplementary Figures.

## Data Availability

The datasets used and/or analyzed during the current study are available from the corresponding author upon reasonable request.
