# Supplementary material for: Label-free multimodal imaging of infected Galleria mellonella larvae
Source: Sci Rep. 2022 Nov 27;12:20416. doi: 10.1038/s41598-022-24846-7 (PMC9701796; doi:10.1038/s41598-022-24846-7)
Supplement: Supplementary file 1 — Supplementary Figures. [file 41598_2022_24846_MOESM1_ESM.docx]

**Supplementary Material**

**Label-free multimodal imaging of infected *Galleria mellonella* larvae**

Elsie Quansah^1,2,+^, Anuradha Ramoji^1,2,3,+,*^, Lara Thieme^4,5^, Kamran Mirza^4,5^, Bianca Goering ^6^, Oliwia Makarewicz^4,5^, Astrid Heutelbeck^6^, Tobias Meyer-Zedler^1,2^, Mathias W. Pletz^3,4^, Michael Schmitt^1,2^ and Jürgen Popp^1,2,3^

^1^ Friedrich-Schiller-University Jena, Institute of Physical Chemistry (IPC) and Abbe Center of Photonics (ACP), Helmholtzweg 4, D-07743 Jena, Germany

^2^ Leibniz Institute of Photonic Technology (IPHT), Member of Leibniz Health Technologies, Albert-Einstein-Straße 9, D-07745 Jena, Germany

^3^ Jena University Hospital, Center for Sepsis Control and Care (CSCC), Friedrich-Schiller-University Jena, Am Klinikum 1, 07747, Jena, Germany

^4^ Jena University Hospital, Institute of Infectious Diseases and Infection Control, Friedrich-Schiller-University Jena, Am Klinikum 1, 07747 Jena, Germany

^5^ Jena University Hospital, Leibniz Center for Photonics in Infection Research, Friedrich Schiller University Jena, 07747 Jena, Germany

^6^ Jena University Hospital, Institute for occupational, social, and environmental medicine, Friedrich-Schiller-University Jena, Am Klinikum 1, 07747, Jena, Germany

*+ Equal contribution*

** Corresponding author: anuradha.ramoji@med.uni-jena.de*

**
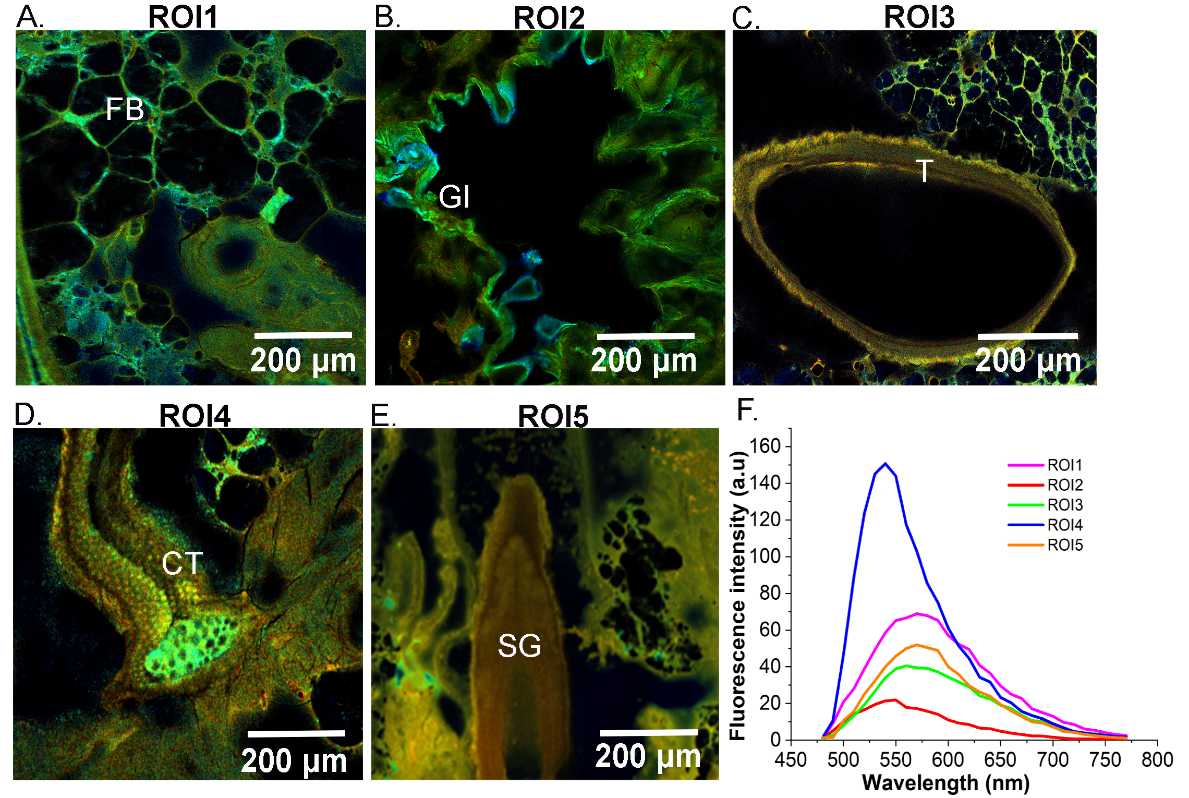
**

**Figure S1**. FLIM images from different ROIs of the control *G. mellonella* larvae tissue depicting (**A**) endogenous fluorophores from the fat body (FB), the crypt from (**B**) the gastrointestinal tract (GI), (**C**) the tracheal (T) wall, (**D**) the cuticle (CT), and the (**E**) silk gland (SG). (**F**) Fluorescence emission spectra from lambda scan were acquired from the ROIs, which correspond to flavins (~540 nm) and lipo-pigments (~570 nm).


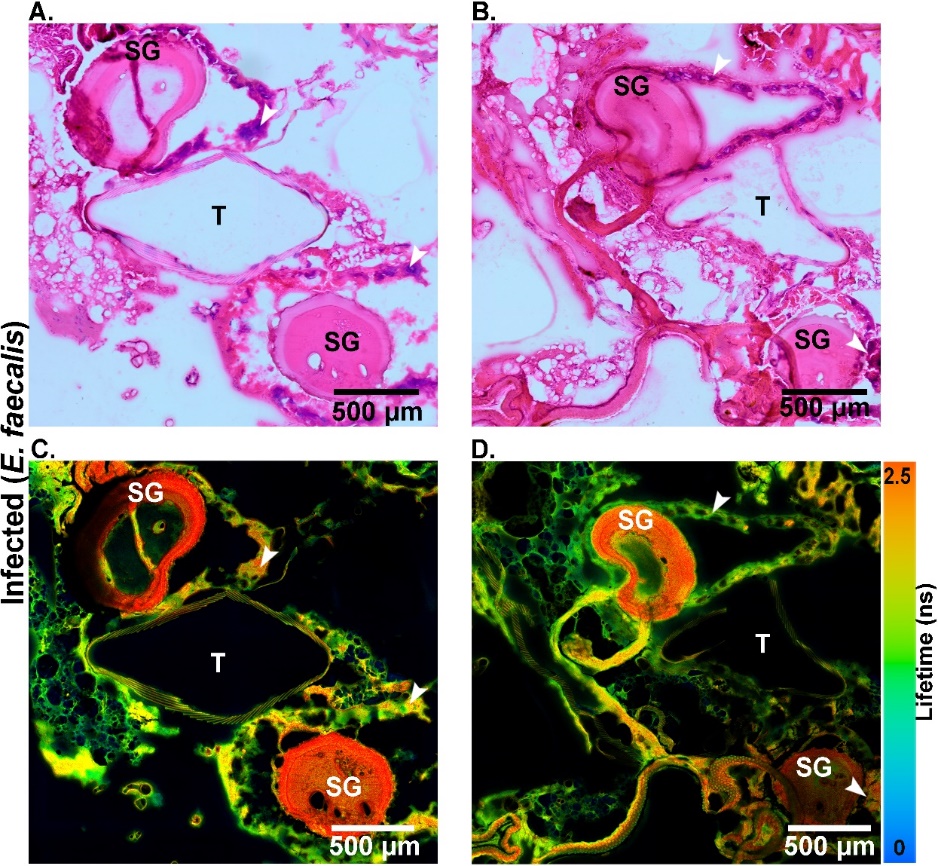


**Figure S2**. Transverse sections of *E. faecalis* infected tissue from *G. mellonella* larvae. **A, B:** HE stained images and **C, D:** FLIM images with lifetime information, indicated as false-color images, ranging from blue to red (scale 0 – 2.5 ns). SG = silk gland, T = trachea, white arrowheads indicate dissociation of the tissue around tubular organs/silk glands as well as basophilic bacterial colonies.


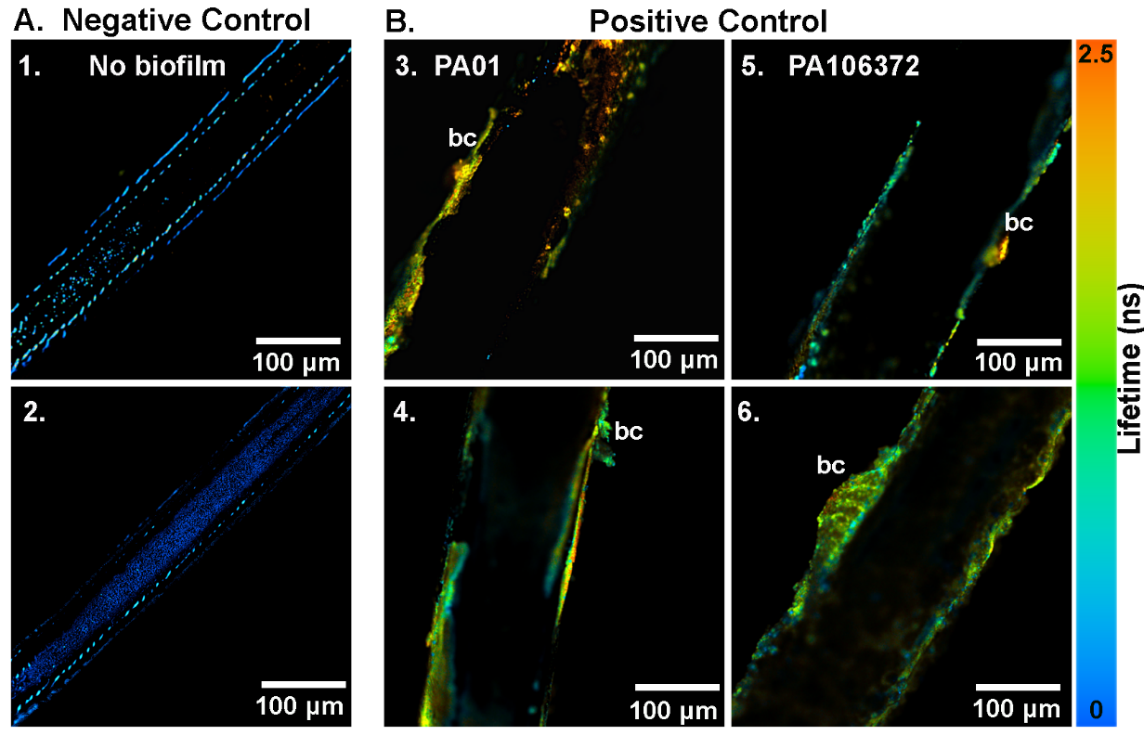


**Figure S3**. FLIM images of the steel implants with biofilm formed by *P. aeruginosa* bacterial strains PA01 and PA106372. The steel implants without biofilm (**A1 and A2**) and with biofilm were formed by the bacterial strains PA01 (**B3 and B4**) and PA106372 (**B5 and B6**). The differing lifetime information is presented as false-color images ranging from blue to red (scale 0 – 2.5 ns).


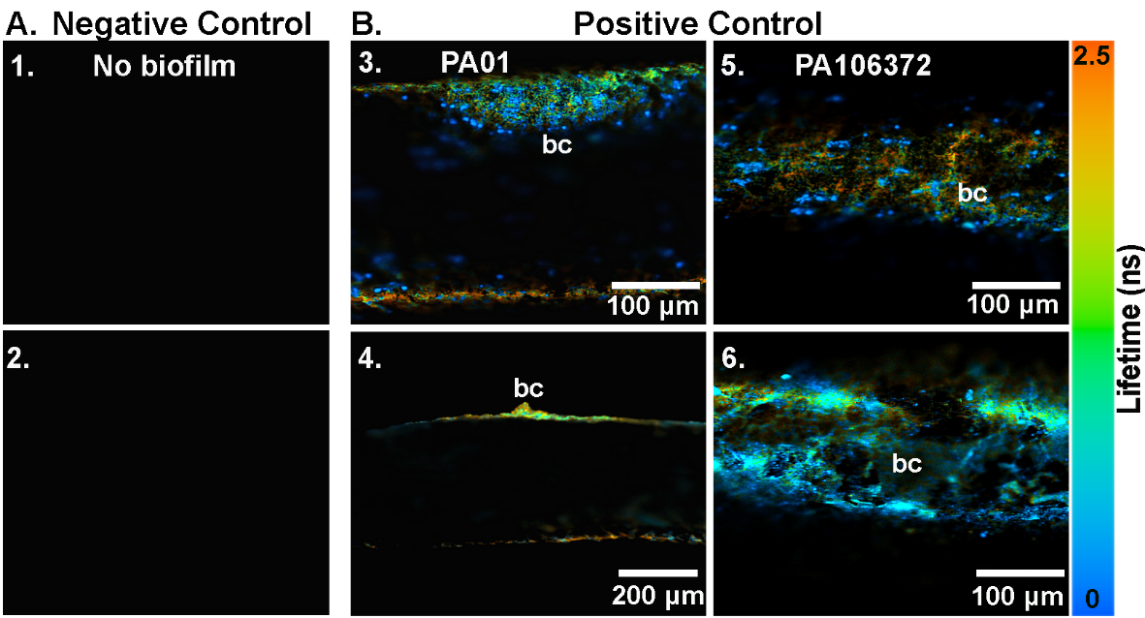


**Figure S4**. FLIM images of the ePTFE implants with biofilm formed by *P. aeruginosa* bacterial strains PA01 and PA106372. The ePTFE implants without biofilm (**A1 and A2**) and with biofilm were formed by the bacterial strains PA01 (**B3 and B4**) and PA106372 (**B5 and B6**). The differing lifetime information is presented as false-color images ranging from blue to red (scale 0 – 2.5 ns).


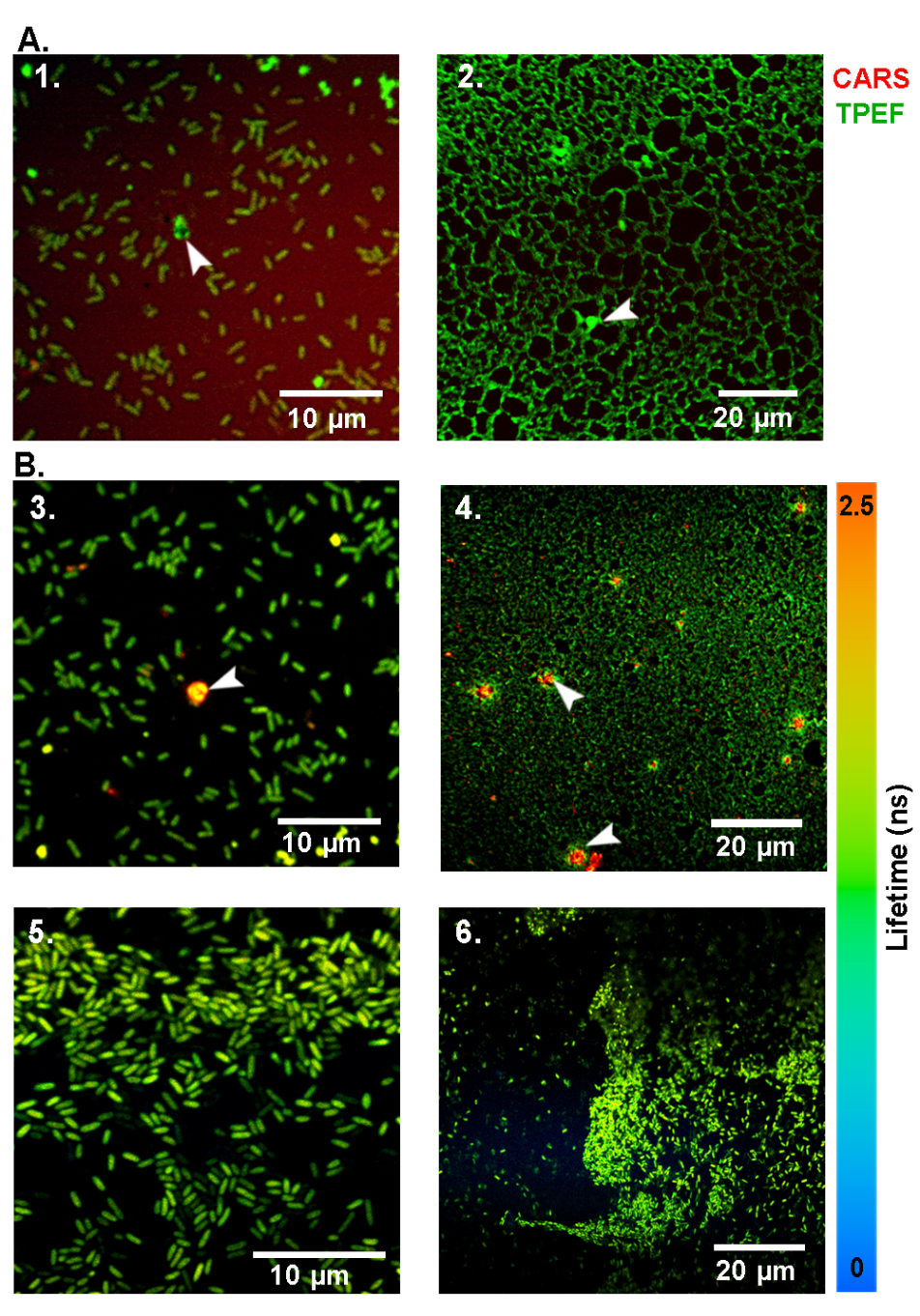


**Figure S5**. The multimodal images (**A**) and the FLIM images (**B**) of pure *P. aeruginosa* bacterial strain PA01 was measured in wet form (**A1**, **B3, and B5**) and dried form (**A2, B4, and B6**). The white arrowheads indicate a cluster of bacteria with high autofluorescence signal. Multimodal images with CARS (red) and TPEF (green) channels are overlayed in false colors. The differing lifetime information is presented as false-color images ranging from blue to red (scale 0 – 2.5 ns).

**
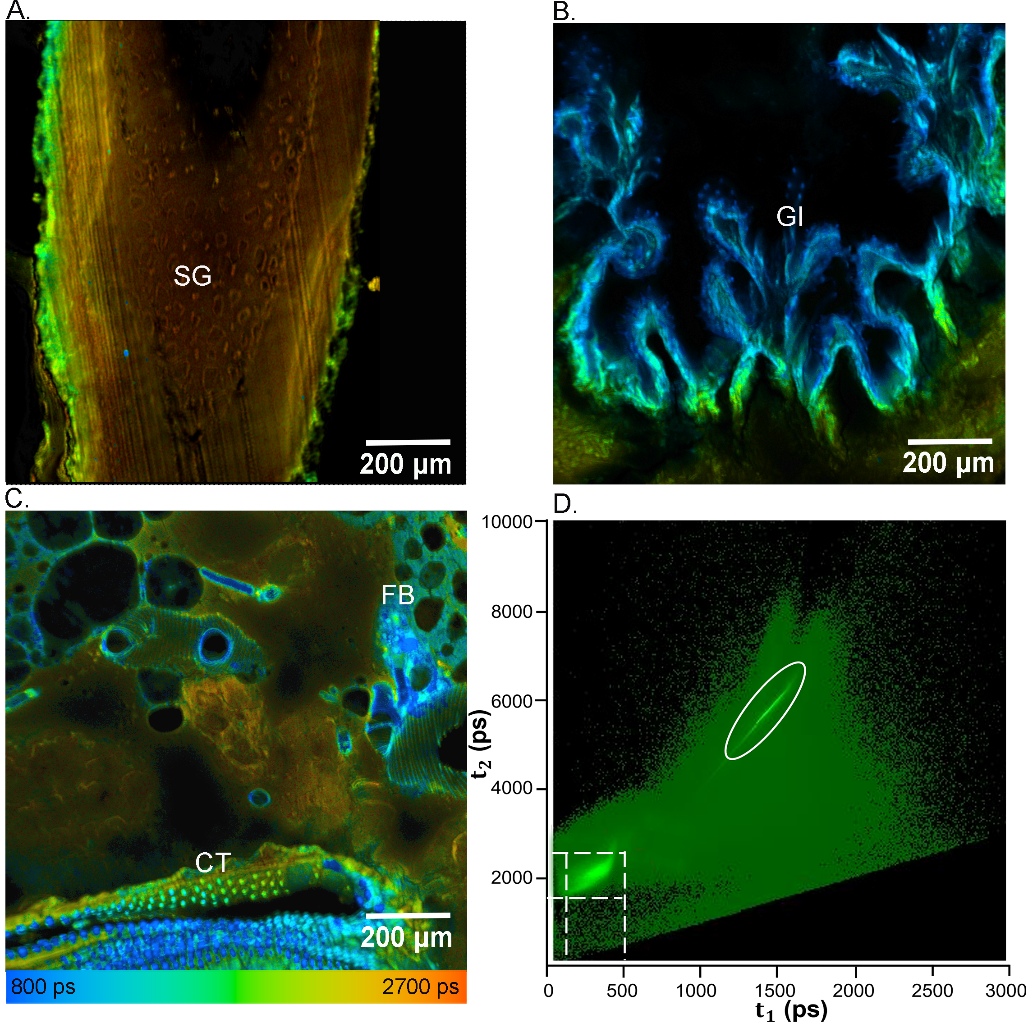
**

**Figure S6**. 2P-FLIM images of the control *G. mellonella* tissue showing NAD(P)H-autofluorescence from (**A**) the silk gland (SG), the crypt from (**B**) the gastrointestinal tract (GI), and (**C**) the cuticle (CT). (**D**) A 2D correlation plot of $t_{1}$ and $t_{2}$ using a biexponential fit of data, which reveals two clusters of the signal. The strongest cluster can be attributed to free and protein-bound NADH, with a short lifetime ($t_{1}$) between 200 – 500 ps and long lifetime component ($t_{2}$) of 1800 -2400 ps. The circled cluster corresponds to other fluorophores when excited at 672 nm.
